# Enhanced cisplatin chemotherapy sensitivity by self-assembled nanoparticles with Olaparib

**DOI:** 10.3389/fbioe.2024.1364975

**Published:** 2024-02-13

**Authors:** Tao Zhang, Xiao Li, Liang Wu, Yue Su, Jiapei Yang, Xinyuan Zhu, Guolin Li

**Affiliations:** ^1^ Key Laboratory of Microecology-immune Regulatory Network and Related Diseases, School of Basic Medicine, Jiamusi University, Jiamusi, China; ^2^ Department of Oral and Maxillofacial Surgery, The First Affiliated Hospital of Harbin Medical University, Harbin, China; ^3^ School of Chemistry and Chemical Engineering, State Key Laboratory of Metal Matrix Composites, Shanghai Jiao Tong University, Shanghai, China; ^4^ Department of Oral, Shanghai Eighth People’s Hospital, Xuhui Branch of Shanghai Sixth People’s Hospital, Shanghai, China

**Keywords:** cisplatin, olaparib, enhanced chemotherapy sensitivity, self-assembly, nanoparticles

## Abstract

Cisplatin (CDDP) is widely used as one kind of chemotherapy drugs in cancer treatment. It functions by interacting with DNA, leading to the DNA damage and subsequent cellular apoptosis. However, the presence of intracellular PARP1 diminishes the anticancer efficacy of CDDP by repairing DNA strands. Olaparib (OLA), a PARP inhibitor, enhances the accumulation of DNA damage by inhibiting its repair. Therefore, the combination of these two drugs enhances the sensitivity of CDDP chemotherapy, leading to improved therapeutic outcomes. Nevertheless, both drugs suffer from poor water solubility and limited tumor targeting capabilities. To address this challenge, we proposed the self-assembly of two drugs, CDDP and OLA, through hydrogen bonding to form stable and uniform nanoparticles. Self-assembled nanoparticles efficiently target tumor cells and selectively release CDDP and OLA within the acidic tumor microenvironment, capitalizing on their respective mechanisms of action for improved anticancer therapy. *In vitro* studies demonstrated that the CDDP-OLA NPs are significantly more effective than CDDP/OLA mixture and CDDP at penetrating cancer cells and suppressing their growth. *In vivo* studies revealed that the nanoparticles specifically accumulated at the tumor site and enhanced the therapeutic efficacy without obvious adverse effects. This approach holds great potential for enhancing the drugs’ water solubility, tumor targeting, bioavailability, and synergistic anticancer effects while minimizing its toxic side effects.

## Introduction

Cancer stands as an imperative global health concern of unparalleled magnitude (Bray et al., 2018; Siegel et al., 2021). The primary treatment methods for cancer currently include surgery, radiation therapy, stem cell therapy (Chen et al., 2022) and chemotherapy. Cisplatin (CDDP) holds a prominent position for cancer therapy due to its extensive utilization (Wang and Lippard, 2005). It operates by engaging with purine bases on DNA, thereby inducing DNA damage, activating diverse signal pathways, and culminating in cellular apoptosis. Nonetheless, the existence of intracellular PARP1 (Poly (ADP-ribose) polymerase 1) plays a pivotal role in identifying and mending DNA strand breaks, consequently impeding the anticancer effectiveness of CDDP (Chaudhuri and Nussenzweig, 2017). Over the span of the preceding four decades, researchers have devoted their efforts to the development of numerous PARP1 inhibitors, among which Olaparib (OLA) stands out as a noteworthy drug (Farmer et al., 2005; Ledermann et al., 2014; Lord and Ashworth, 2017). As a PARP1 inhibitor, OLA augments the accumulation of DNA damage by impeding its repair. Based on the mechanism of action of drugs, OLA can enhance the anticancer sensitivity of CDDP. Therefore, the synergistic utilization of CDDP and OLA emerges as a promising and more effective treatment approach. This strategic alliance between CDDP and OLA holds great potential in improving cancer treatment efficacy (Prasad et al., 2017). However, prevailing combination therapies predominantly rely on a rudimentary physical blend of two chemotherapeutic drugs. The contrasting physical and chemical attributes of two drugs, coupled with their distinct pharmacokinetic profiles, posed a formidable challenge in maintaining an equitable distribution ratio within tumor tissue. Moreover, both CDDP and OLA exhibit limited water solubility and lack tumor targeting capabilities, further impeding the realization of optimal outcomes in combination therapy.

In recent years, the field of combined cancer therapy has witnessed widespread utilization of nanotechnology-based drug delivery systems, aiming to reduce drug toxicity and enhance drug bioavailability (Zhao et al., 2020; Yi et al., 2022; Jiang et al., 2023; Wang et al., 2023). These systems included liposomes (Liu et al., 2022), polymer nanoparticles, and hydrogels. For instance, Yuan et al. designed polyethylene glycol-modified nano-liposomes to facilitate the simultaneous delivery of docetaxel and resveratrol (Zhang et al., 2022). Wang and Li developed MPPD@IR825/DTX NPs, which utilized a pH-responsive charge-reversal mechanism to enable targeted delivery of docetaxel and a photosensitizer (IR825), enabling chemo-photothermal combination therapy (Wang et al., 2022). Li, Jin, and their research team created a nano-vaccine that responds to the tumor microenvironment. The vaccine was based on biodegradable hollow manganese dioxide and enabled synergistic cancer therapy (Wang et al., 2022). Additionally, Li and colleagues designed an injectable pH-responsive OE peptide hydrogel as a carrier material for the anticancer drugs gemcitabine and paclitaxel (Liu et al., 2022). These endeavors successfully addressed the issue of limited utilization of chemotherapeutic drugs caused by their poor water solubility. They also enhanced the tumor targeting abilities of drugs and mitigated toxic side effects on healthy organs. However, it is crucial to note that nano-scale drug delivery systems heavily relied on the use of exogenous carriers, which posed challenges in clinical applications. Limited drug loading efficiency and the complex metabolic, degradation, and excretion processes of these carriers within the body increased the potential risk of adverse reactions related to toxicity or the immune system.

**SCHEME 1:**
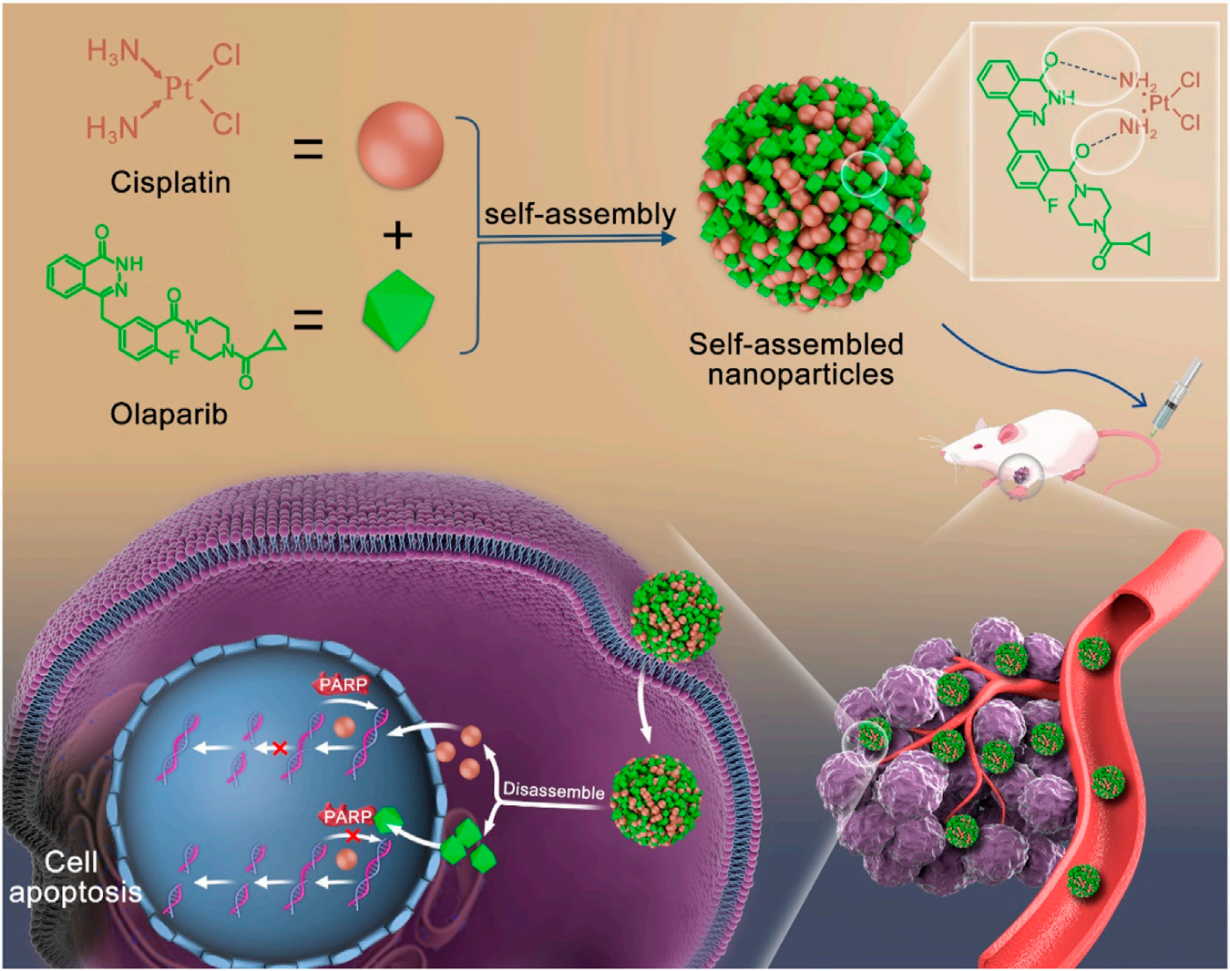
Schematic diagram of self-assembly route of CDDP-OLA nanoparticles and their synergistic mechanism.

To address these issues, the carrier-free strategy has been developed as a suitable choice. Carrier-free self-assembly strategy enhanced drug targeting to tumors, improved drug efficacy, mitigated the toxic side effects of chemotherapy drugs, and avoided the risks associated with carriers (Song et al., 2012; Huang et al., 2014; Zhang et al., 2015; Zhu et al., 2020). This strategy utilized intermolecular interactions for self-assembly into defined nanostructures (Zhang et al., 2017; Wu et al., 2018). Based on the chemical structures of CDDP and OLA, as well as the enhanced the CDDP chemotherapy sensitivity by OLA, we proposed the self-assembly of CDDP and OLA through hydrogen bonding to form stable and homogeneous nanoparticles (CDDP-OLA NPs). This nanosizing strategy improved the aqueous solubility of both drugs, eliminated the need for exogenous carriers and enabled targeted delivery to the tumor site through enhanced permeability and retention (EPR) effects. These enhancements resulted in improved properties that facilitated apoptosis in cancer cells while minimized harmful effects on normal cells. The acidic tumor microenvironment disrupted hydrogen bonding, leading to the liberation of free CDDP and OLA. CDDP, with its ability to induce DNA damage, initiated the apoptotic process, while OLA augmented the anticancer sensitivity of CDDP by inhibiting PARP activity, thereby reducing the repair of damaged DNA. Due to their mechanisms of action, it is highly plausible that the combination of these two drugs within nanoparticles can enhance efficacy against cancer, while minimize the toxic side effects associated with chemotherapy drugs (Scheme 1).

## Experimental

### Materials

All reagents used in this study were commercially available and did not undergo any additional purification. CDDP and OLA were obtained from Meilun Biotech (Dalian, China). Dimethyl sulfoxide (DMSO) was supplied by Sinopharm Chemical Reagent Co., Ltd. (Shanghai, China). Phosphate Buffered Saline (PBS), Dulbecco’s Modified Eagle’s Medium (DMEM), Fetal Bovine Serum (FBS), 0.25% pancreatin, and penicillin-streptomycin (100×) were acquired from Gibco (Grand Island, USA). 3-(4,5-dimethyl-2-thiazolyl)-2,5-diphenyl-2H-tetrazolium bromide (MTT), Annexin V-FITC/PI apoptosis detection kits, cell cycle test kits, and mitochondrial membrane potential (MMP) detection kits were obtained from Beyotime Biotechnology Co., LTD. (Shanghai, China). Hoechst live cell stain was acquired from Sigma Aldrich Co. (St Louis, MO, USA). All antibodies used for Western blotting were purchased from Affinity Biosciences (Jiangsu, China), and the protein ladder was obtained from ThermoFisher Scientific (Waltham, MA, USA). Cell experiment consumables were sourced from Corning Incorporated (Corning, NY, USA).

### Preparation of CDDP-OLA NPs

To fabricate CDDP-OLA NPs, CDDP (2.98 mg, 1 mmol) and OLA (4.34 mg, 1 mmol) were individually dissolved in 20 µL of DMSO. Subsequently, the solutions of CDDP and OLA were combined and added to 2 mL of ultrapure water under stirring, followed by an additional 10 min of stirring. To eliminate DMSO and unbound drug, we conducted the purification of CDDP-OLA NPs through ultrafiltration centrifugation. The ultrafiltration process employed a molecular weight cut-off of 500, and the centrifugation was carried out at 4°C for 10 min at 4,500 rpm.

### Characterization of CDDP-OLA NPs

A total of 10 µL of freshly made CDDP-OLA NPs was dropped onto copper grids with a carbon film and left to stand for 10 min. A transmission electron microscope (Tecnai G2 Spirit Biotwin; FEI, Hillsboro, OR, USA) was utilized, with the voltage set at 120 kV, to image the dimensions and form of CDDP-OLA NPs. Dynamic light scattering (DLS; Zetasizer Nano ZS90, Malvern, England) was utilized at a temperature of 25°C to evaluate the size distribution of CDDP-OLA NPs in hydrodynamic fluid with 2 mL of the solution in disposable plastic cuvettes. The FT-IR spectra of CDDP-OLA NPs at 303, 333, 363, 393 and 423 K were determined using an FT-IR spectrometer (Nicolet 6700, ThermoFisher Scientific, Waltham, MA, USA). The scanning parameters were as follows: scan frequency range, 400–4,000 cm^−1^; resolution, 4.0 cm^−1^; and scan times, 32. A 400 MHz nuclear magnetic resonance (NMR) spectrometer (Avance III 400 MHz, Bruker, Germany) was used to measure the ^1^H NMR spectra at various temperatures: 303, 333, 363, and 393 K. Deuterium dimethylsulphoxide (DMSO-*d*_6_) was used to dissolve the samples. With an ultraviolet (UV)-visible (Vis) Spectrophotometer (UV-1800, Shimadzu, Japan), measurements in the UV-Vis spectrum were performed using cuvettes with a 1-cm light path. The platinum (Pt) atom was detected using an AXIS UltraDLD instrument (Kratos, Japan).

### All-atom molecular dynamics (MD) simulation

MD simulation was conducted using the LAMMPS programming package (Wang et al., 2004) to investigate the self-assembly behavior of OLA and CDDP in an aqueous solution. The simulation employed a time step of 1 fs. The simulation systems were constructed using DFF (Plimpton, 1995), comprising 2,000 OLA molecules, 2,000 CDDP molecules, and 40,000 water molecules. The initial 5 ns simulation run was performed at a temperature of 298.15 K and a pressure of 1 atm. Subsequently, the simulation was extended for an additional 10 ns, during which trajectory data were collected for analysis. The all-atom MD simulation utilized the TEAM force field, and the parameters for CDDP were adopted from previous publications (Yao et al., 1994; Cundari et al., 1996; Jin et al., 2015). The cross interactions between different atom types were modelled using Lorentz-Berthelot mixing rules. A tail correction was applied to limit the van der Waals (vdW) interactions within 1.2 nm, while long-range interactions within the all-atom force field were calculated using the particle-mesh Ewald (PME) method. Temperature was controlled using a Nosé–Hoover thermostat (Sebesta et al., 2016), and pressure was regulated using a barostat. VMD software was employed for visualizing the simulated boxes (Nosé, 1984). The structure of the aqueous solution was characterized using the radial distribution function (RDF) $g_{ab}\left(r_{ab}\right)$ , which represents the average distribution between two atoms with types a and b over the MD trajectory,

$$g_{ab}\left(r_{ab}\right)=\frac{\rho_{ab}\left(r_{ab}\right)}{N_{b}/V}$$

where $N_{b}=\int_{V}\rho_{ab}\left(r\right)\text{dr}$ . RDF $g_{ab}\left(r_{ab}\right)$ demonstrates the local correlations between two types of atoms with separation $r_{ab}$ .

### Cell culture

DMEM enhanced with 10% inactivated FBS, 1% 100 U/mL penicillin, and 100 μg/mL streptomycin was used to cultivate MDA-MB-231 cells at 37°C.

### Cellular uptake of CDDP-OLA NPs

To gain insights into the cellular uptake of NPs, co-assembled Cy5.5 NPs were prepared. Briefly, Cy5.5-loaded CDDP-OLA NPs were generated by adding Cy5.5, dissolved in DMSO, to the NPs solution while stirring. *In vitro* uptake of the CDDP-OLA NPs was assessed using laser-scanning confocal microscopy (LSCM) and flow cytometry. MDA-MB-231 cells were seeded in 6-well plates at a density of 5 × 10^5^ cells per well for flow cytometry experiments. The cells were then exposed to CDDP-OLA NPs for 0.5, 1, 2, 4, and 6 h. Following trypsin treatment and three washes with PBS, the uptake behavior of CDDP-OLA NPs was analyzed using a flow cytometer (LSRFortessa, BD, Franklin Lakes, NJ, USA). For LSCM investigations, MDA-MB-231 cells were seeded on glass coverslips placed in 6-well plates. Each well contained 3 × 10^5^ cells. Subsequently, the cells were exposed to CDDP-OLA NPs for 0.5, 1, 2, 4, and 6 h. After three cold PBS rinses, the cells were fixed with 4% paraformaldehyde. Nuclei were stained with Hoechst for 5 min. Glass coverslips were mounted onto slides, and the uptake behavior of CDDP-OLA NPs was examined using a LSCM (TCS SP8 STED 3X, Leica, Germany).

### Cytotoxicity of CDDP-OLA NPs

We evaluated *in vitro* cytotoxicity via MTT assays. A total of 1.5 × 10^4^ MDA-MB-231 cells were seeded into each well using 96-well plates. Different doses (2, 4, 8, 16, 32, 64, and 128 µM) of CDDP, OLA, CDDP/OLA mixture, and CDDP-OLA NPs were applied to the cells for 48 h. Control cells were grown in DMEM. After 48 h, each well received 20 µL of a 5 mg/mL MTT solution in PBS. Followed a 4 h incubation in the dark at 37°C and removal of the supernatant, 150 µL of DMSO was added to each well to dissolve the formazan crystals. Followed 10 min of shaking, a BioTek Synergy H4 hybrid reader was used to measure the absorbance at 490 nm. The following equation was used to determine cell viability:

$$Cell\;survival=\frac{{OD}_{490\;sample}-{\;OD}_{490\;blank}}{{OD}_{490\;control}-\;{\;OD}_{490\;blank}}\times 100$$

where ${OD}_{490\;sample}$ , ${OD}_{490\;control}$ , and ${\;OD}_{490\;blank}$ are the ultraviolet absorbance degrees at 490 nm with drugs, without drugs, and with DMEM alone.

### Cell apoptosis

Cell apoptosis was analyzed via flow cytometry. Using a 6-well plate, 5 × 10^5^ MDA-MB-231 cells were seeded into each well. After cell adhesion, cells were incubated with the same concentrations of CDDP, OLA, CDDP/OLA mixture, and CDDP-OLA NPs (CDDP 25 μM, OLA 25 µM) for 48 h. Cells were separated using pancreatin after 48 h, then washed three times with fresh PBS before added a binding buffer. The cell suspension was then stained with 10 µL PI and 5 µL Alexa Fluor FITC-coupled annexin-V. These processed cells were analyzed on the FITC and PI channels on a flow cytometer.

### Mitochondrial membrane potential (MMP) measurement

Using the fluorescent probe JC-1, the MMP of the cells was determined. MDA-MB-231 cells were seeded in a 6-well plate (5 × 10^5^ cells each well). After cell adhesion, cells were incubated with the same concentration of CDDP, OLA, CDDP/OLA mixture, and CDDP-OLA NPs (CDDP 25 μM, OLA 25 µM) for 48 h. Followed three rounds of rinsing with cold PBS, cells were treated with JC-1 (1.2×) for 20 min at 37°C. Afterwards, JC-1 dye buffer (1×) was used to rinse the cells twice to stain the mitochondria. Hoechst was used to stain cell nuclei for 5 min. The MMP was then assessed using a LSCM.

### Cell cycle analysis

Using flow cytometry, cell cycle analysis was carried out. Briefly, 5 × 10^5^ MDA-MB-231 cells were seeded into each well of a 6-well plate. After cell adhesion, all cells were incubated with same concentrations of CDDP, OLA, CDDP/OLA mixture, and CDDP-OLA NPs (CDDP 20 μM, OLA 20 µM) for 48 h. After 48 h detached cells were gathered and mixed with adherent cells digested via trypsinisation. Followed medium wash, the cells were pelleted, resuspended in 70% ethanol, and kept at 4°C for 1 h. Followed a centrifugation, the cells were washed three times with PBS, resuspended in a PI dye solution, and then incubated for 30 min at 37°C without light. Cell cycle analysis was performed using a flow cytometer. Using doublet discrimination, the percentage of cells in each cell cycle phase was ascertained from 5 × 10^4^ cells. FlowJo software was used to analyze the cell cycle position.

### Western blot analysis

In 100 mm culture dishes, MDA-MB-231 cells were seeded at a density of 5 × 10^6^ cells in 10 mL DMEM complete medium. After adhesion, cells were cultivated with same concentrations of CDDP, OLA, CDDP/OLA mixture, and CDDP-OLA NPs (CDDP 25 μM, OLA 25 µM) for 24 h. Cells were then washed three times with cool PBS and disrupted in radioimmunoprecipitation assay (RIPA) lysis buffer to extract cellular proteins. A bicinchoninic acid (BCA) protein assay kit was used to determine the amount of protein present in the extracts. Protein was run on an SDS-PAGE gel in equal proportions, transferred to 0.45 µm polyvinylidene fluoride (PVDF) membranes (Millipore, USA), and then blocked in 5% skim milk in Tris-buffered saline with Tween (TBST). Afterwards, mouse anti-human GAPDH, p53, and PARP1, and rabbit anti-human c-Caspase-3, Bax, and Bcl-2 antibodies were incubated with the membranes. GADPH was employed as a control for loading. A ChemiDocTM MP imaging system (Bio-Rad, Hercules, CA, USA) was used to visualize the protein bands.

### Tumor models

Female Balb/c nude mice (6 weeks, Certificate No. 20221017Abbbbbbbb0105000315) were obtained from Ziyuan Experimental Animal Technology Co., LTD. (Hangzhou, China). In order to conduct an *in vivo* anticancer efficacy test, mice were given a hypodermic injection in the right flank containing 200 µL of a cell solution comprising 5 × 10^6^ MDA-MB-231 cells. Experiments began when the tumors got to be approximately 100 mm^3^ in size.

### *In vivo* tumor targeting and biodistribution study

Two groups were randomly selected from mice with MDA-MB-231 tumors. Through the tail vein, mice were administered 200 µL of either Cy5.5-loaded CDDP-OLA NPs or Cy5.5 alone. Cy5.5 intrinsic fluorescence was measured at 0, 1, 4, 8, 12, and 24 h after injection using a Kodak multimode imaging system. Moreover, to assess the distribution of CDDP-OLA NPs *in vivo*, an injection of CDDP-OLA NPs solution (5 mg CDDP/kg, 7 mg OLA/kg) was given to MDA-MB-231 tumor-bearing mice through the tail vein at different time points. The heart, liver, spleen, lung, kidneys, and tumor were among the principal tissues dissected for fluorescence imaging.

### Evaluation of antitumor effects

Twenty-five nude mice bearing MDA-MB-231 tumors were randomly assigned to one of five groups. Intravenous injections of PBS, CDDP, OLA, CDDP/OLA mixture, and CDDP-OLA NPs (CDDP 5 mg/kg, OLA 7 mg/kg) were administered to tumor-bearing animals once every 3 days for a total of 21 days. Before each drug delivery, the tumor weight and volume were calculated using a vernier caliper and platform scale. The following formula was used to determine the tumor volume:

$$Volume\;\left({mm}^{3}\right)=\frac{1}{2}\times length\;\left(mm\right)\times width\left({mm}^{2}\right)$$

Mice in all groups were sacrificed after 21 days, following the IACUC protocol. We removed, weighed, and photographed their organs, which included heart, liver, spleen, lung, kidneys, and tumors. Prior to being embedded in paraffin, the tumors and other tissues underwent further dissection and were then preserved in 4% polyoxymethylene. The tumors were then chopped into pieces for Hematoxylin and Eosin (H&E) staining, Ki67 labeling, and TUNEL assay. H&E staining was applied to sections of additional organs.

### Statistical analysis

Data were collected from a minimum of three separate replicates. Individual data points were compared using the Student’s t-test. *p* < 0.05 was considered statistically significant in each case.

## Results and discussion

### Preparation and characterizations of CDDP-OLA NPs

**FIGURE 1:**
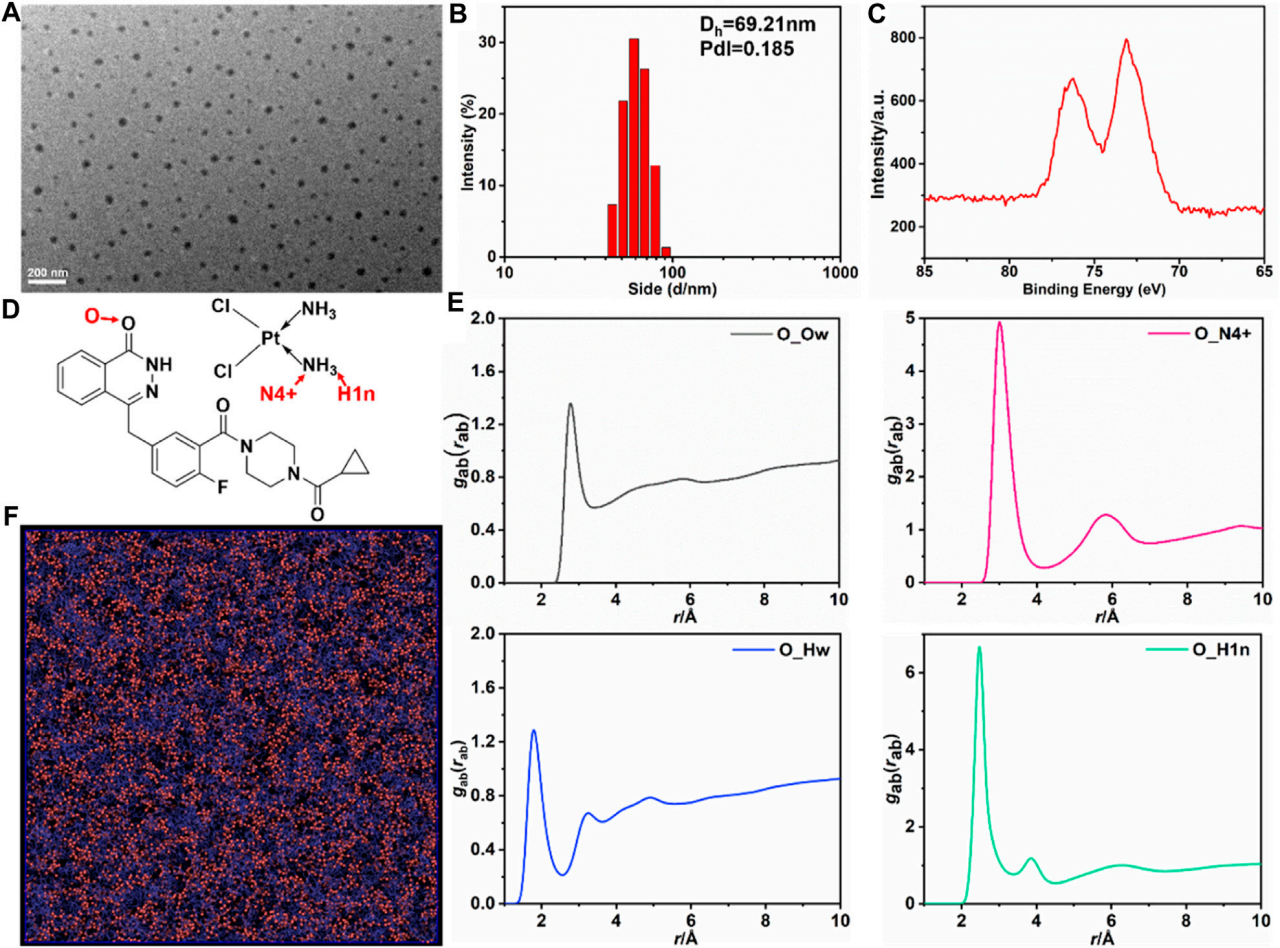
Characterizations of the CDDP-OLA NPs and All-atom MD simulation of CDDP and OLA in aqueous solution. **(A)** TEM image of CDDP-OLA NPs. Scale bar = 200 nm. **(B)** Hydrodynamic particle size of CDDP-OLA NPs in PBS. **(C)** XPS of detected NPs. **(D)** Atom type definitions in OLA and CDDP. **(E)** Radial distribution functions $g_{ab}\left(r_{ab}\right)$ , atom type O represents the carbonyl group in the OLA, type N4+ and H1n are the nitrogen and hydrogen in NH3 of CDDP, and Ow and Hw are the oxygen and hydrogen atoms of water molecules. **(F)** Snapshot of molecular configuration where OLA is purple, CDDP is red, and water molecules are not shown for clarity.

In our current study, we provided evidence for the self-assembly of the chemotherapeutic drugs CDDP and the PARP inhibitor OLA into NPs through a slow mixing process. Previous research (Humphrey et al., 1996; Liu et al., 2016; Zhang et al., 2016) led us to hypothesize that intermolecular interactions, such as hydrogen bonding, between the two molecules contribute to the formation of CDDP-OLA NPs. The size and shape of the resulting CDDP-OLA NPs were characterized via dynamic light scattering (DLS) and transmission electron microscopy (TEM). The hydrodynamic size of the NPs was approximately 60 nm, while the diameter observed under TEM imaging was around 50 nm. These size parameters, in conjunction with the enhanced permeability and retention effect, make the NPs favorable for accumulation at tumor sites, as depicted in Figures 1A, B. To further confirm the successful construction of CDDP-OLA NPs, the presence of platinum atoms was characterized using X-ray photoelectron spectroscopy (XPS) (Figure 1C).

To elucidate the mechanisms underlying the self-assembly of CDDP-OLA NPs and investigate the presence of intermolecular interactions between the two drug compounds, we employed various techniques. Firstly, we examined the existence of hydrogen bonding through ^1^H NMR spectra and FTIR spectroscopy at different temperatures. Previous reports (Ma et al., 2016) have shown that breaking hydrogen bonds can significantly affect the movement of related protons. As the temperature increased from 303 K to 393 K, the hydrogen of the NH group in OLA gradually shifted to a lower magnetic field (from 12.56 to 12.08 ppm), indicating the presence of hydrogen bonding (Supplementary Figures S1A–C). Furthermore, the FT-IR spectra demonstrated that the stretching vibration peak of the C=O bond shifted to a higher wavenumber indicating the involvement of the C=O bond in hydrogen bond formation (Supplementary Figures S2A–F). These results confirmed the interaction between CDDP and OLA through hydrogen bonding.

### Molecular simulations to interrogate the self-assembly dynamics and mechanisms of CDDP-OLA NPs

To obtain a comprehensive understanding of CDDP and OLA self-assembly in aqueous solution, all-atom simulation was performed, with the simulation system containing 2,000 OLA, 2,000 CDDP, and 40,000 water molecules. A TEAM force field was adopted to describe the atomic interactions; the parameters of CDDP were entered based on previous publications (Yao et al., 1994; Cundari et al., 1996; Jin et al., 2015). The total 15 ns MD simulation was performed, and the last 10 ns trajectory was used for analysis. OLA interacted with CDDP to form aggregates in the aqueous solution. Atom type definitions in the OLA and CDDP are shown in Figure 1D. The RDF between OLA, CDDP, and water indicated that the self-assembly structure resulted from competition between intermolecular interactions among the three species. The carbonyl group in OLA strongly interacted with the NH3 group in CDDP, as illustrated by the high peaks in the RDF of the pairs O-N4+ and O-H1n pairs, located at 2.7 Å (O-H1n) and 3.0 Å (O-N4+), corresponding to the hydrogen bonding (Figure 1E). The association between the drugs and water was less favored and the peak in RDF fluctuated with average densities. The hydrogen bonding between OLA and CDDP was visualized in Figure 1F, clearly showing that CDDP binds closely with OLA.

### Cellular uptake

**FIGURE 2:**
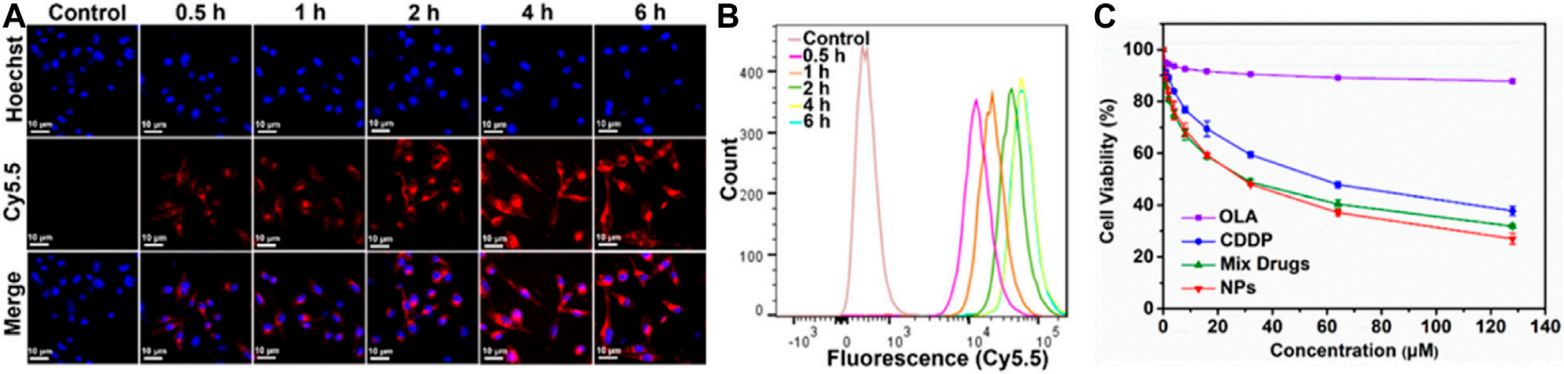
Effect of CDDP-OLA NPs on cancer cellular uptake, and cell proliferation *in vitro*. **(A)** Confocal images of MDA-MB-231 cells containing CDDP-OLA NPs at 0.5, 1, 2, 4, and 6 h. Blue represents the Hoechst nuclear stain and red represents Cy5.5. Scale bar = 10 µm. **(B)** Analysis of MDA-MB-231 cells using flow cytometry after they were exposed to CDDP-OLA NPs for 0.5, 1, 2, 4, and 6 h. **(C)** Viability of MDA-MB-231 cells cultured for 48 h with varying doses of CDDP, OLA, CDDP/OLA mixture, and CDDP-OLA NPs.

To determine whether the CDDP-OLA NPs could be taken up by MDA-MB-231 cells, cellular uptake was investigated via LSCM and flow cytometry. Confocal imaging demonstrated that Cy5.5 fluorescence intensity in MDA-MB-231 cells steadily rose during the course of the incubation period (Figure 2A), which was consistent with our flow cytometry data (Figure 2B; Supplementary Figure S3). These results demonstrated that CDDP-OLA NPs could be effectively taken up by MDA-MB-231 cells.

### *In vitro* cytotoxicity

The *in vitro* cytotoxicity of different drug formulations was assessed using the MTT test. CDDP, OLA, CDDP/OLA mixture, and CDDP-OLA NPs were each applied to MDA-MB-231 cells in varying doses. The negative control cells that had not been treated. Followed a 48-h incubation period, both the CDDP/OLA mixture and the CDDP-OLA NPs exhibited higher cytotoxicity than the free CDDP with MDA-MB-231 cells, demonstrated that OLA effectively improves the oncological outcome of CDDP. As shown in Figure 2C and Supplementary Figure S4, the IC_50_ values of CDDP and the CDDP/OLA mixture in MDA-MB-231 cells were 55.14 and 30.08 µM, respectively. Meanwhile, the IC_50_ of CDDP-OLA NPs was 27.72 µM. It is important to note that the CDDP/OLA mixture showed inferior efficacy compared to the NPs due to the latter’s higher cellular internalization capability and greater bioavailability (Lin et al., 2010).

### Effects of CDDP-OLA NPs on the cell cycle

**FIGURE 3:**
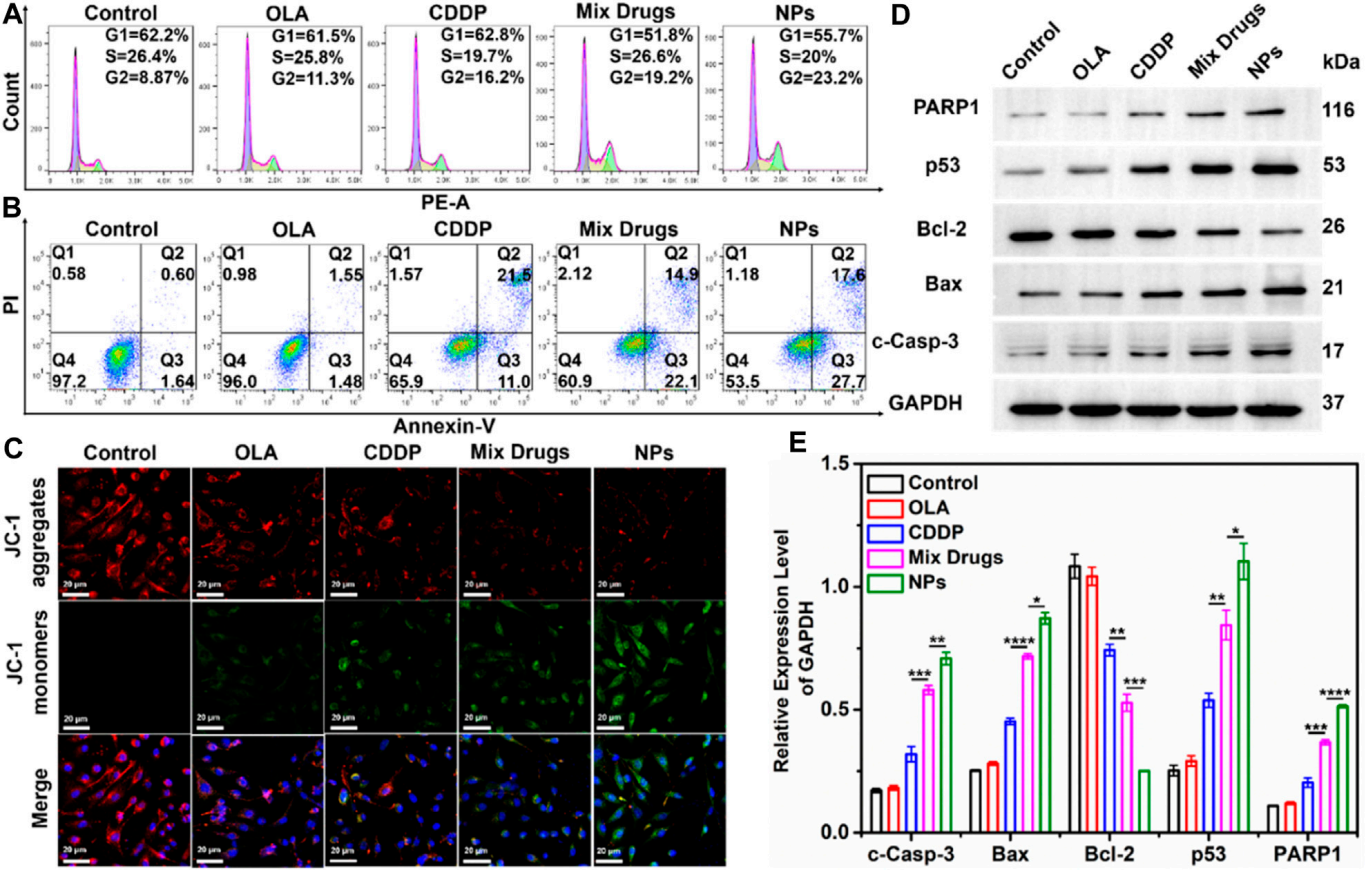
Effect of CDDP-OLA NPs on cancer cell cycle, cell apoptosis, mitochondrial membrane potential (∆Ψm), and cell apoptosis-related protein expression *in vitro*. **(A)** Cell cycle distribution of MDA-MB-231 cells treated with PBS, OLA, CDDP, CDDP/OLA mixture, and CDDP-OLA NPs. **(B)** Apoptosis of MDA-MB-231 cells stimulated by PBS, CDDP, OLA, CDDP/OLA mixture, and CDDP-OLA NPs. **(C)** Mitochondrial damage in MDA-MB-231 cells stimulated by PBS, CDDP, OLA, CDDP/OLA mixture, and CDDP-OLA NPs, as determined via LSCM. Hoechst-stained nuclei are shown in blue, red represents JC-1 aggregates, and green represents JC-1 monomers. Scale bar = 20 µm. **(D)** Expression and **(E)** relative protein levels of c-Caspase-3, Bax, Bcl-2, p53, and PARP in MDA-MB-231 cells treated with OLA, CDDP, CDDP/OLA mixture, and CDDP-OLA NPs (OLA 20 µM and CDDP 20 µM). *p < 0.05, **p < 0.01, ***p < 0.001, ****p < 0.0001 indicated significantly different between two groups.

The cell cycle distributions of MDA-MB-231 cells incubated with CDDP, OLA, CDDP/OLA mixture, and CDDP-OLA NPs are shown in Figure 3A. All treatments induced an increase in the fraction of cells in G2 compared to the controls. Further, the cells exposed to CDDP-OLA NPs exhibited the highest proportion of G2 phase cells (23.2%) among all the groups. These results indicated that CDDP-OLA NPs could induce G2 phase arrest and subsequent apoptosis to a greater extent than CDDP, or CDDP/OLA mixture.

### Apoptosis of cancer cells induced by CDDP-OLA NPs

We evaluated the capacity of CDDP-OLA NPs for inducing tumor cell apoptosis. We treated MDA-MB-231 cells with CDDP, OLA, CDDP/OLA mixture, or CDDP-OLA NPs for 48 h, then we double-stained the cells with FITC and PI. As shown in Figure 3B, the percentages of apoptotic MDA-MB-231 cells after treatment were 65.9, 60.9, and 53.5% with CDDP, CDDP/OLA mixture, and CDDP-OLA NPs treatment, respectively. This could have been due to the CDDP-OLA NPs being successfully internalized and retained in MDA-MB-231 cells, while the drugs that were free or mixed could leave cells with ease. Such elevated drug accumulation in tumor cells has been shown to cause greater apoptosis (Maeda et al., 2000). The aforementioned findings indicated that NPs have a greater capacity to induce apoptosis than a single agent or mixed drugs.

### Analysis of mitochondrial damage and apoptosis mechanism

Mitochondrial damage in cancer cells was assessed using JC-1 dye, which built up in healthy mitochondria and released red fluorescence. This dye inversely dispersed into a monomeric state to produce green fluorescence in response to a drop in the mitochondrial membrane potential (∆Ψm). As seen in Figure 3C, after separated 48 h treatments with PBS, CDDP, and OLA, high red fluorescence and faint green fluorescence were observed, indicative of healthy mitochondria. In contrast, strong green fluorescence and modest red fluorescence were seen in the cells after treatment with the CDDP/OLA mixture and CDDP-OLA NPs, particularly in the latter group, suggested considerable damage to the mitochondria.

### Mechanism of action of the CDDP-OLA NPs

While the CDDP-OLA NPs exhibited superior *in vitro* cytotoxicity compared to either free drug or the mixture of both, their mechanism of action remained unclear. We sought to obtain greater insight into the mechanism of CDDP-OLA NPs action using Western blot analysis. In this study, the expression of p53 protein was markedly elevated by CDDP-OLA NPs compared to the single drug and control treatment; the same trend was observed with the expression of Bax protein, but the opposite was observed with Bcl-2 protein. In addition, the expression levels of c-Caspase-3 and PARP1 proteins were increased in MDA-MB-231 cells exposed to CDDP-OLA NPs compared to the other four groups (Figures 3D, E). These results were repeated three times and can be seen in the Supplementary Material (Supplementary Figure S5).

**SCHEME 2:**
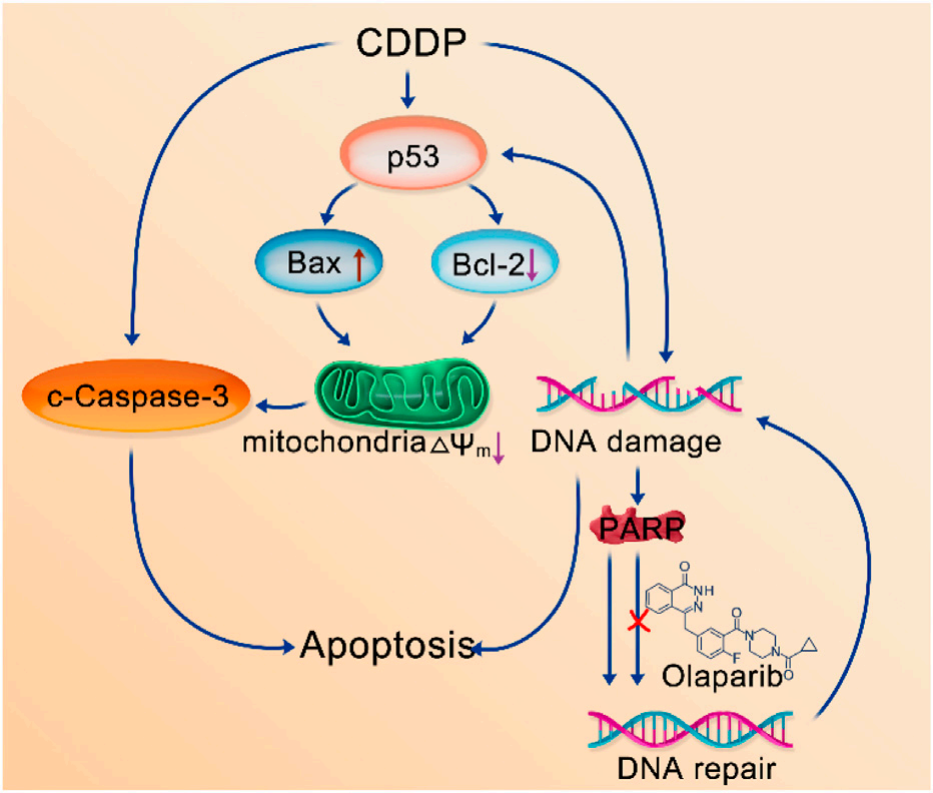
Mechanisms of the combination of CDDP and OLA-induced MDA-MB-231 cell apoptosis.

In general, the caspase signaling pathway serves as a crucial apoptotic marker; the induction of apoptosis leads to caspase activation (Savitskaya and Onishchenko, 2015). PARP1 is the main substrate of caspase-3, being cleaved by it upon the initiation of apoptosis. As a result of this cleavage, PARP1 is no longer able to carry out its role in genome maintenance. DNA damage can activate the p53 signaling pathway (Polo and Jackson, 2011). Further, p53 proteins are linked to oxidative stress. Mitochondria play a pivotal role in the apoptotic cascade (Lin et al., 2018), and downstream proteins, including Bax and Bcl-2, can cause cancer cell apoptosis by destroying the mitochondria (Deus et al., 2015). Based on the aforementioned studies and our experimental proposed that CDDP-OLA NPs released CDDP and OLA after aggregated at the tumor site. CDDP could induce DNA damage in tumor cells and activated the caspase signaling pathway to trigger apoptosis. Additionally, it could activate the p53 signaling pathway, and the downstream mitochondria-related genes, Bax and Bcl-2, regulated by p53, could be up and downregulated, respectively. This leaded to a further decrease in the mitochondrial membrane potential (∆Ψm) and regulated caspase-dependent apoptosis. Meanwhile, OLA, as a PARP inhibitor, hinders DNA repair, which further amplified DNA damage, leading to the induction of apoptosis (Scheme 2).

### Real-time optical imaging and biodistribution analysis

**FIGURE 4:**
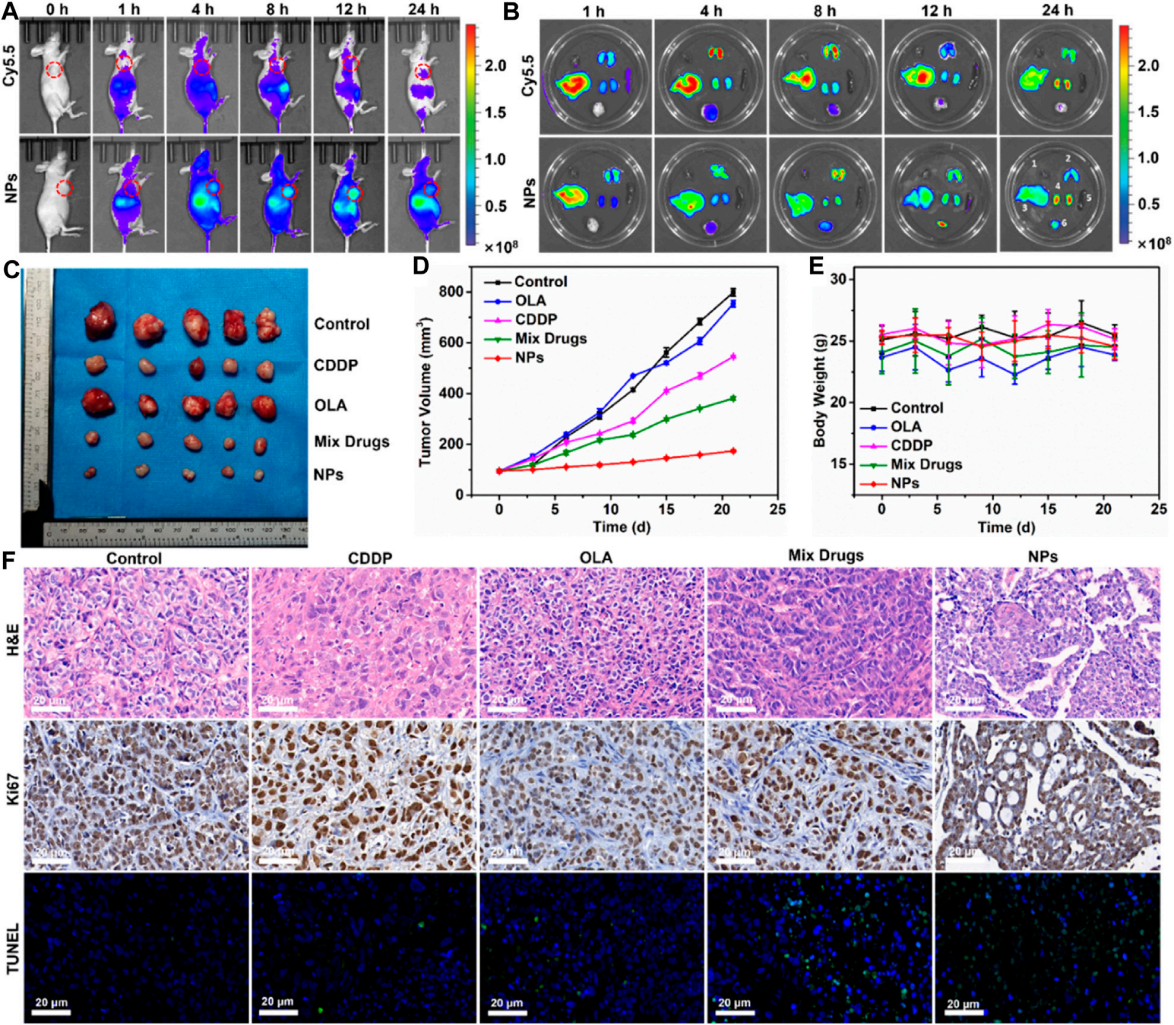
Antitumor efficacy of CDDP-OLA NPs in MDA-MB-231 tumor-bearing Balb-c/nude mice. **(A)** Free Cy5.5 and Cy5.5-loaded CDDP-OLA NPs imaging *in vivo*. **(B)** *In vivo* biodistribution of the two aforementioned formulations—1: heart; 2: lung; 3: liver; 4: kidneys; 5: spleen; and 6: tumor. **(C)** Tumor tissue after 21 days of therapy. **(D)** Treatment-related changes in tumor volume. **(E)** Treatment-related changes in animal weight. **(F)** H&E, Ki67, and TUNEL-stained slices of tumor tissue.

Based on the strong antitumor efficacy of CDDP-OLA NPs observed *in vitro*, we assessed their antitumor impact *in vivo*. First, the tumor-specific targeting capability of CDDP-OLA NPs was evaluated using *in vivo* imaging of nude mice carrying MDA-MB-231 tumors. At 1, 4, 8, 12, and 24 h after injection, free Cy5.5 and NPs loaded with Cy5.5 were examined. As illustrated in Figure 4A, the brightest fluorescence signal from tumors was observed at 4 h following the injection of free Cy5.5, after which the fluorescence intensity declined. Since free Cy5.5 is quickly degraded *in vivo*, at 12 h after injection, the fluorescent signal of free Cy5.5 in MDA-MB-231 tumor-bearing nude mice was nearly undetectable. Meanwhile, the fluorescence signals of Cy5.5-loaded CDDP-OLA NPs near the tumor site steadily increased over the first 12 h, which may be attributed to the EPR effect causing CDDP-OLA NPs to accumulate there. Biodistribution analysis in several organs consistently indicated that free Cy5.5 was completely cleared during the first 12 h post-injection, while CDDP-OLA NPs continued to accumulate at the tumor site (Figure 4B). These data highlight the improved tumor-targeting capacity of CDDP-OLA NPs compared to the free small-molecule drugs.

### *In vivo* antitumor efficacy

With the enhanced biodistribution and tumor accumulation of NPs confirmed, we proceeded to evaluate their antitumor efficacy *in vivo*. A tail vein intravenous infusion of PBS (control), CDDP, OLA, CDDP/OLA mixture, or CDDP-OLA NPs was administered to mice with MDA-MB-231 tumors. As shown in Figures 4C, D, the tumor diameters in mice given PBS injections increased from around 100 mm^3^ to approximately 800 mm^3^ through the course of therapy, with no tumor suppression observed. When mice were given CDDP or OLA alone, tumor volumes increased to approximately 550 mm^3^ and 750 mm^3^, respectively, proving that a single anticancer treatment only moderately limit tumor growth. Owing to the synergistic effects of CDDP and OLA in suppressing MDA-MB-231 cell growth, tumor volumes in the CDDP/OLA mixture group reached approximately 380 mm^3^. Meanwhile, mice administered CDDP-OLA NPs experienced minor tumor growth to approximately 170 mm^3^, proved the superior efficacy of this formulation. We attributed this effect to the enhanced biodistribution and effective tumor accumulation. There was no discernible difference in body weight among the mice with MDA-MB-231 tumors in the early part of the experimental period, but slight increases in weight were observed at the later stages (Figure 4E). This was attributed to the small doses of administered therapeutics.

The antitumor efficacy of CDDP-OLA NPs was further analyzed via H&E staining, Ki67, and TUNEL assays. H&E staining revealed an abundance of cancer cells in tumor tissue from control mice, without obvious signs of damage. In contrast, tumor tissue from groups treated with CDDP-OLA NPs contained a high level of cells devoid of nuclei. In addition, a considerable reduction in the number of brown (Ki67-positive) tumor cells was observed in CDDP-OLA NPs-treated mice as opposed to those who received the different formulas. TUNEL analysis indicated that CDDP-OLA NPs induced tumor cell apoptosis most potently (Figure 4F). Importantly, heart, liver, spleen, lung, and kidney tissue from NPs-treated mice exhibited no obvious injury (Supplementary Figure S6). Taken together, these results indicated that CDDP-OLA NPs exhibit promising antitumor efficacy and favorable tolerability.

In current work, we developed a novel kind of supramolecular self-delivery nanodrugs with OLA using non-covalent interactions for enhancing CDDP chemotherapy sensitivity. This nanodrug system was formulated by the hydrogen bonding interaction and hydrophobic interaction of OLA and CDDP, which avoids chemical modification of parent drugs or introduction of any non-clinically proven materials and thus facilitates their clinical translation. Both the computer simulations and experiments confirmed the feasibility of their self-assembly. These supramolecular nanodrugs exhibited a synergistic therapeutic effect by inhibiting the DNA repair and increased DNA damage accumulation and enhancing the sensitivity of CDDP chemotherapy. More importantly, *in vitro* and *in vivo* studies demonstrated that these nanodrugs improved pharmacokinetics, bioavailability and therapeutic efficacy while having considerably reduced side effects of free drugs.

## Conclusion

The complementary mechanisms of CDDP and OLA synergistically enhance the sensitivity of CDDP chemotherapy, resulted in improved therapeutic outcomes. CDDP induced DNA damage for apoptosis, while OLA, a PARP1 inhibitor, inhibited DNA repair and increased DNA damage accumulation. The synergistic use of CDDP and OLA showed promise for more effective cancer treatment. By self-assembling CDDP and OLA into nanoparticles (CDDP-OLA NPs) through hydrogen bonding, with a uniform shape and size distribution, the CDDP-OLA NPs exhibited improved cell uptake, superior tumor selectivity, and pronounced therapeutic efficacy *in vitro* and *in vivo* compared to CDDP or CDDP/OLA mixture. We enhanced both chemotherapeutic agents' solubility and tumor targeting without the need for external carriers. This optimization improved bioavailability, maximized the anticancer effect while minimized the toxic side effects of chemotherapy. Importantly, the CDDP-OLA NPs were easily prepared without the requirement for sophisticated chemical changes. Considering these benefits, we believe that this strategy holds great potential as an anticancer therapy with a low risk of adverse effects.

## Data Availability

The original contributions presented in the study are included in the article/Supplementary Material, further inquiries can be directed to the corresponding authors.

## Abbreviations

BCA, bicinchoninic acid; CDDP, Cisplatin; CDDP-OLA NPs, Cisplatin-Olaparib nanoparticles; DLS, dynamic light scattering; DMEM, Dulbecco’s Modified Eagle’s Medium; DMSO, Dimethyl sulfoxide; DMSO-d6, Deuterium dimethylsulphoxide; EPR, enhanced permeability and retention; FT-IR, Fourier transform infrared; FBS, Fetal Bovine Serum; H&E, Hematoxylin and Eosin; LSCM, laser-scanning confocal microscopy; MD, molecular dynamics; MMP, mitochondrial membrane potential; MTT, 3-(4,5-dimethyl-2-thiazolyl)-2,5-diphenyl-2H-tetrazolium bromide; NMR, nuclear magnetic resonance; NPs, nanoparticles; OLA, Olaparib; PARP1, Poly (ADP-ribose) polymerase 1; PBS, Phosphate Buffered Saline; Pt, platinum; PME, particle-mesh Ewald; PVDF, polyvinylidene fluoride; RIPA, radioimmunoprecipitation assay; RDF, radial distribution function; TBST, Tris-buffered saline with Tween; TEM, transmission electron microscope; UV, ultraviolet; vdW, van der Waals; Vis, visible; XPS, X-ray photoelectron spectroscopy.

## References

[B1] Bray F. Ferlay J. Soerjomataram I. Siegel R. L. Torre L. A. Jemal A. (2018). Global cancer statistics 2018: GLOBOCAN estimates of incidence and mortality worldwide for 36 cancers in 185 countries. CA Cancer J. Clin. 68 (6), 394–424. 10.3322/caac.21492 30207593

[B2] Chaudhuri A. R. Nussenzweig A. (2017). The multifaceted roles of PARP1 in DNA repair and chromatin remodelling. Nat. Rev. Mol. Cell. Biol. 18 (10), 610–621. 10.1038/nrm.2017.53 28676700 PMC6591728

[B3] Chen J. Li D. Li H. Zhu K. Shi L. Fu X. (2022). Cell membrane-targeting NIR fluorescent probes with large Stokes shifts for ultralong-term transplanted neural stem cell tracking. Front. Bioeng. Biotechnol. 11, 1139668. 10.3389/fbioe.2023.1139668 PMC9948019 36845195

[B4] Cundari T. R. Fu W. Moody E. W. Slavin L. L. Snyder L. A. Sommerer S. O. (1996). Molecular mechanics force field for platinum coordination complexes. J. Phys. Chem. 100 (46), 18057–18064. 10.1021/jp961240x

[B5] Deus C. M. Zehowski C. Nordgren K. Wallace K. B. Skildum A. Oliveira P. J. (2015). Stimulating basal mitochondrial respiration decreases doxorubicin apoptotic signaling in H9c2 cardiomyoblasts. Toxicology 334, 1–11. 10.1016/j.tox.2015.05.001 25997894

[B6] Farmer H. McCabe N. Lord C. J. Tutt A. N. J. Johnson D. A. Richardson T. B. (2005). Targeting the DNA repair defect in BRCA mutant cells as a therapeutic strategy. Nature 434 (7035), 917–921. 10.1038/nature03445 15829967

[B7] Huang P. Wang D. Su Y. Huang W. Zhou Y. Cui D. (2014). Combination of small molecule prodrug and nanodrug delivery: amphiphilic drug-drug conjugate for cancer therapy. J. Am. Chem. Soc. 136 (33), 11748–11756. 10.1021/ja505212y 25078892

[B8] Humphrey H. Dalke A. Schulten K. (1996). VMD: visual molecular dynamics. J. Mol. Graph 14 (1), 33–38. 10.1016/0263-7855(96)00018-5 8744570

[B9] Jiang M. Liao J. Liu C. Liu J. Chen P. Zhou J. (2023). Metal-organic frameworks/metal nanoparticles as smart nanosensing interfaces for electrochemical sensors applications: a mini-review. Front. Bioeng. Biotechnol. 11, 1251713. 10.3389/fbioe.2023.1251713 37614634 PMC10442806

[B10] Jin Z. Yang C. Cao F. Li F. Jing Z. Chen L. (2015). Hierarchical atom type definitions and extensible all-atom force fields. J. Comput. Chem. 37 (7), 653–664. 10.1002/jcc.24244 26537332

[B11] Ledermann J. Harter P. Gourley C. Friedlander M. Vergote I. Rustin G. (2014). Olaparib maintenance therapy in patients with platinum-sensitive relapsed serous ovarian cancer: a preplanned retrospective analysis of outcomes by BRCA status in a randomised phase 2 trial. Lancet Oncol. 15 (8), 852–861. 10.1016/S1470-2045(14)70228-1 24882434

[B12] Lin I. H. Cheng C. C. Yen Y. C. Chang F. C. (2010). Synthesis and assembly behavior of heteronucleobase-functionalized poly(ε-caprolactone). Macromolecules 43 (3), 1245–1252. 10.1021/ma9026614

[B13] Lin Y. Shen Z. Song X. Liu X. Yao K. (2018). Comparative transcriptomic analysis reveals adriamycin-induced apoptosis via p53 signaling pathway in retinal pigment epithelial cells. J. Zhejiang Univ. Sci. B 19 (12), 895–909. 10.1631/jzus.B1800408 30507074 PMC6305253

[B14] Liu K. Xing R. Zou Q. Ma G. Möhwald H. Yan X. (2016). Simple peptide-tuned self-assembly of photosensitizers towards anticancer photodynamic therapy. Angew. Chem. Int. Ed. Engl. 55 (9), 3036–3039. 10.1002/anie.201509810 26804551

[B15] Liu Y. Quan X. Li J. Huo J. Li X. Zhao Z. (2022). Liposomes embedded with PEGylated iron oxide nanoparticles enable ferroptosis and combination therapy in cancer. Natl. Sci. Rev. 10 (1), nwac167. 10.1093/nsr/nwac167 36684514 PMC9843134

[B16] Liu Y. Ran Y. Ge Y. Raza F. Li S. Zafar H. (2022). pH-Sensitive peptide hydrogels as a combination drug delivery system for cancer treatment. Pharmaceutics 14 (3), 652. 10.3390/pharmaceutics14030652 35336026 PMC8948763

[B17] Lord C. J. Ashworth A. (2017). PARP inhibitors: synthetic lethality in the clinic. Science 355 (6330), 1152–1158. 10.1126/science.aam7344 28302823 PMC6175050

[B18] Ma K. Xing R. Jiao T. Shen G. Chen C. Li J. (2016). Injectable self-assembled dipeptide-based nanocarriers for tumor delivery and effective *in vivo* photodynamic therapy. ACS Appl. Mater Interfaces 8 (45), 30759–30767. 10.1021/acsami.6b10754 27778498

[B19] Maeda H. Wu J. Sawa T. Matsumura Y. Hori K. (2000). Tumor vascular permeability and the EPR effect in macromolecular therapeutics: a review. J. Control Release 65 (1-2), 271–284. 10.1016/s0168-3659(99)00248-5 10699287

[B20] Nosé S. (1984). A unified formulation of the constant temperature molecular dynamics methods. J. Chem. Phys. 81 (1), 511–519. 10.1063/1.447334

[B21] Plimpton S. (1995). Fast parallel algorithms for short-range molecular dynamics. J. Comput. Phys. 117 (1), 1–19. 10.1006/JCPH.1995.1039

[B22] Polo S. E. Jackson S. P. (2011). Dynamics of DNA damage response proteins at DNA breaks: a focus on protein modifications. Genes. Dev. 25 (5), 409–433. 10.1101/gad.2021311 21363960 PMC3049283

[B23] Prasad C. B. Prasad S. B. Yadav S. S. Pandey L. K. Singh S. Pradhan S. (2017). Olaparib modulates DNA repair efficiency, sensitizes cervical cancer cells to cisplatin and exhibits anti-metastatic property. Sci. Rep. 7 (1), 12876. 10.1038/s41598-017-13232-3 28993682 PMC5634505

[B24] Savitskaya M. A. Onishchenko G. E. (2015). Mechanisms of apoptosis. Biochem. (Mosc) 80 (11), 1393–1405. 10.1134/S0006297915110012 26615431

[B25] Sebesta F. Sláma V. Melcr J. Futera Z. Burda J. V. (2016). Estimation of transition-metal empirical parameters for molecular mechanical force fields. J. Chem. Theory Comput. 12 (8), 3681–3688. 10.1021/acs.jctc.6b00416 27337427

[B26] Siegel R. L. Miller K. D. Fuchs H. E. Jemal A. (2021). Cancer statistics, 2021. CA Cancer J. Clin. 71 (1), 7–33. 10.3322/caac.21654 33433946

[B27] Song J. Zhou J. Duan H. (2012). Self-assembled plasmonic vesicles of SERS-encoded amphiphilic gold nanoparticles for cancer cell targeting and traceable intracellular drug delivery. J. Am. Chem. Soc. 134 (32), 13458–13469. 10.1021/ja305154a 22831389

[B28] Wang C. Yang X. Qiu H. Huang K. Xu Q. Zhou B. (2023). A co-delivery system based on chlorin e6-loaded ROS-sensitive polymeric prodrug with self-amplified drug release to enhance the efficacy of combination therapy for breast tumor cells. Front. Bioeng. Biotechnol. 11, 1168192. 10.3389/fbioe.2023.1168192 37064246 PMC10090272

[B29] Wang D. Lippard S. J. (2005). Cellular processing of platinum anticancer drugs. Nat. Rev. Drug Discov. 4 (4), 307–320. 10.1038/nrd1691 15789122

[B30] Wang J. Wolf R. M. Caldwell J. W. Kollman P. A. Case D. A. (2004). Development and testing of a general amber force field. J. Comput. Chem. 25 (9), 1157–1174. 10.1002/jcc.20035 15116359

[B31] Wang W. Chen X. Li J. Jin Q. Jin H. J. Li X. (2022). Hollow MnO2 nanoparticles loaded with functional genes as nanovaccines for synergistic cancer therapy. ACS Appl. Nano Mat. 5, 10537–10547. 10.1021/acsanm.2c01877

[B32] Wang X. Gu Y. Li Q. Xu Y. Shi Y. Wang Z. (2022). Synergistic chemo-photothermal cancer therapy of pH-responsive polymeric nanoparticles loaded IR825 and DTX with charge-reversal property. Colloids Surf. B Biointerfaces 209 (Pt 2), 112164. 10.1016/j.colsurfb.2021.112164 34735859

[B33] Wu C. Xu L. Shi L. Gao X. Li J. Zhu X. (2018). Supramolecularly self-assembled nano-twin drug for reversing multidrug resistance. Biomater. Sci. 6 (8), 2261–2269. 10.1039/c8bm00437d 29999073

[B34] Yao S. Plastaras J. P. Marzilli L. G. (1994). A molecular mechanics AMBER-type force field for modeling platinum complexes of guanine derivatives. Inorg. Chem. 33 (26), 6061–6077. 10.1021/ic00104a015

[B35] Yi X. Zeng W. Wang C. Chen Y. Zheng L. Zhu X. (2022). A step-by-step multiple stimuli-responsive metal-phenolic network prodrug nanoparticles for chemotherapy. Nano Res. 15, 1205–1212. 10.1007/s12274-021-3626-2

[B36] Zhang L. Lin Z. Chen Y. Gao D. Wang P. Lin Y. (2022). Co-delivery of Docetaxel and Resveratrol by liposomes synergistically boosts antitumor efficiency against prostate cancer. Eur. J. Pharm. Sci. 174, 106199. 10.1016/j.ejps.2022.106199 35533965

[B37] Zhang R. Xing R. Jiao T. Ma K. Chen C. Ma G. (2016). Carrier-free, chemophotodynamic dual nanodrugs via self-assembly for synergistic antitumor therapy. ACS Appl. Mater Interfaces 8 (21), 13262–13269. 10.1021/acsami.6b02416 27176934

[B38] Zhang T. Huang P. Shi L. Su Y. Zhou L. Zhu X. (2015). Self-assembled nanoparticles of amphiphilic twin drug from floxuridine and bendamustine for cancer therapy. Mol. Pharm. 12 (7), 2328–2336. 10.1021/acs.molpharmaceut.5b00005 25996874

[B39] Zhang Z. Shi L. Wu C. Su Y. Qian J. Deng H. (2017). Construction of a supramolecular drug-drug delivery system for non-small-cell lung cancer therapy. ACS Appl. Mater Interfaces 9 (35), 29505–29514. 10.1021/acsami.7b07565 28809468

[B40] Zhao M. Straten D. v. Broekman M. L. D. Préat V. Schiffelers R. M. (2020). Nanocarrier-based drug combination therapy for glioblastoma. Theranostics 10 (3), 1355–1372. 10.7150/thno.38147 31938069 PMC6956816

[B41] Zhu L. Guo Y. Qian Q. Yan D. Li Y. Zhu X. (2020). Carrier-free delivery of precise drug-chemogene conjugates for synergistic treatment of drug-resistant cancer. Angew. Chem. Int. Ed. Engl. 59 (41), 17944–17950. 10.1002/anie.202006895 32643224

