# Supplementary material for: Enhanced cisplatin chemotherapy sensitivity by self-assembled nanoparticles with Olaparib
Source: Front Bioeng Biotechnol. 2024 Feb 13;12:1364975. doi: 10.3389/fbioe.2024.1364975 (PMC10898354; doi:10.3389/fbioe.2024.1364975)
Supplement: Supplementary file 1 [file DataSheet1.pdf]

Enhanced Cisplatin Chemotherapy Sensitivity by Self-assembled Nanoparticles with Olaparib

Tao Zhang,[1] Xiao Li,[2] Liang Wu,[3] Yue Su[3] Jiapei Yang,[3] Xinyuan Zhu*[3] and Guolin Li*[1,4]

1 Key Laboratory of Microecology-immune Regulatory Network and Related Diseases, School of Basic Medicine, Jiamusi University, Jiamusi, Heilongjiang 154000, P.R. China

2 Department of Oral and Maxillofacial Surgery, The First Affiliated Hospital of Harbin Medical University, 23 Youzheng Street, Nangang District, Harbin 150001, P.R. China

3 School of Chemistry and Chemical Engineering, State Key Laboratory of Metal Matrix Composites, Shanghai Jiao Tong University, 800 Dongchuan Road, Shanghai 200240, P.R. China

4 Department of Oral, Shanghai Eighth People's Hospital, Xuhui Branch of Shanghai Sixth People's Hospital, Shanghai 200235, China.

* **Corresponding author:** Xinyuan Zhu, School of Chemistry and Chemical Engineering, State Key Laboratory of Metal Matrix Composites, Shanghai Jiao Tong University, 800 Dongchuan Road, Shanghai 200240, P.R. China Email: xyzhu@sjtu.edu.cn

Guolin Li, Key Laboratory of Microecology-immune Regulatory Network and Related Diseases, School of Basic Medicine, Jiamusi University, Jiamusi, Heilongjiang 154000, P.R. China. Email: liguolin@jmsu.edu.cn

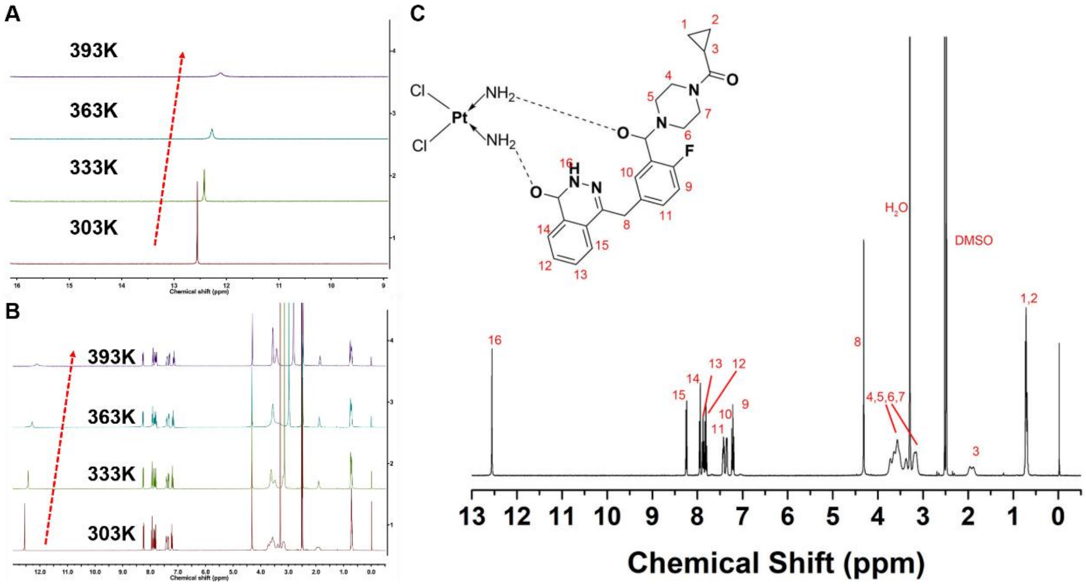

Figure S1 (A) ^{1}H-NMR NH chemical shift of the CDDP-OLA NPs as a function of temperature in DMSO-d_6. (B) ^{1}H-NMR chemical shift of the CDDP-OLA NPs as a function of temperature in DMSO-d_6. (C) ^{1}H-NMR of CDDP-OLA NPs at 303 K in DMSO-d_6.

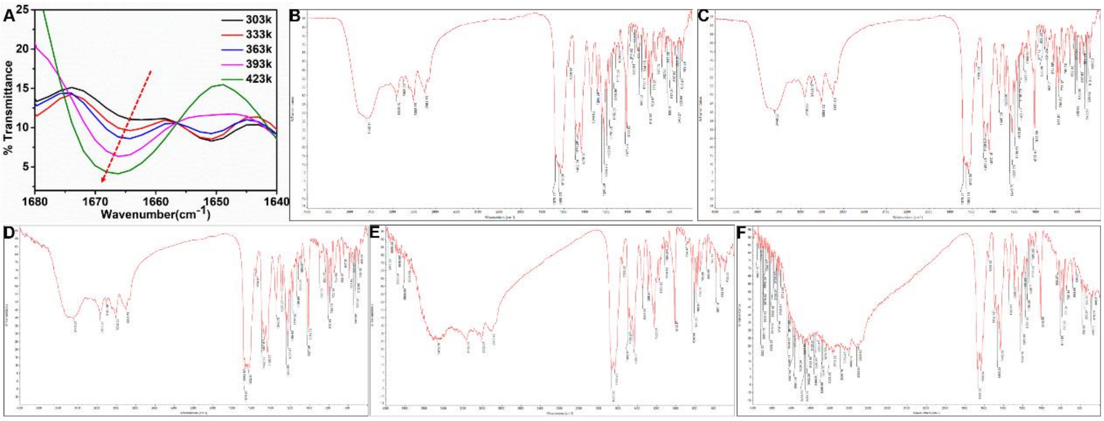

Figure S2 (A) Variable temperature combinations (B) 303K (C) 333K (D) 363K (E) 393K and (F) 423K FTIR spectra of CDDP-OLA NPs. Samples were allowed to equilibrate for 10 min at each temperature.

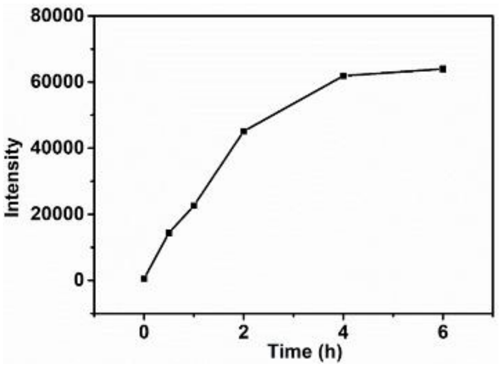

Figure S3 The cellular uptake of CDDP-OLA NPs by MDA-MB-231 cells. Fluorescence of the intracellular Cy5.5 was used to quantify the internalization efficiency of CDDP-OLA NPs as determined by flow cytometry.

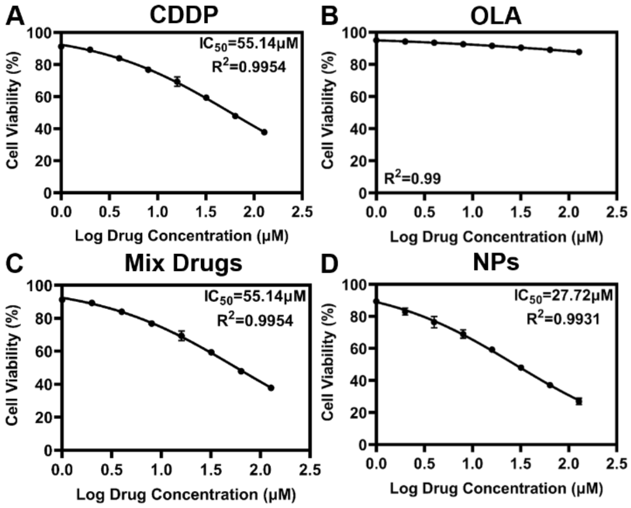

Figure S4 IC_{50} curves of (A) CDDP; (B) OLA; (C) Mix Drugs; (D) CDDP-OLA NPs for MDA-MB-231 cells.

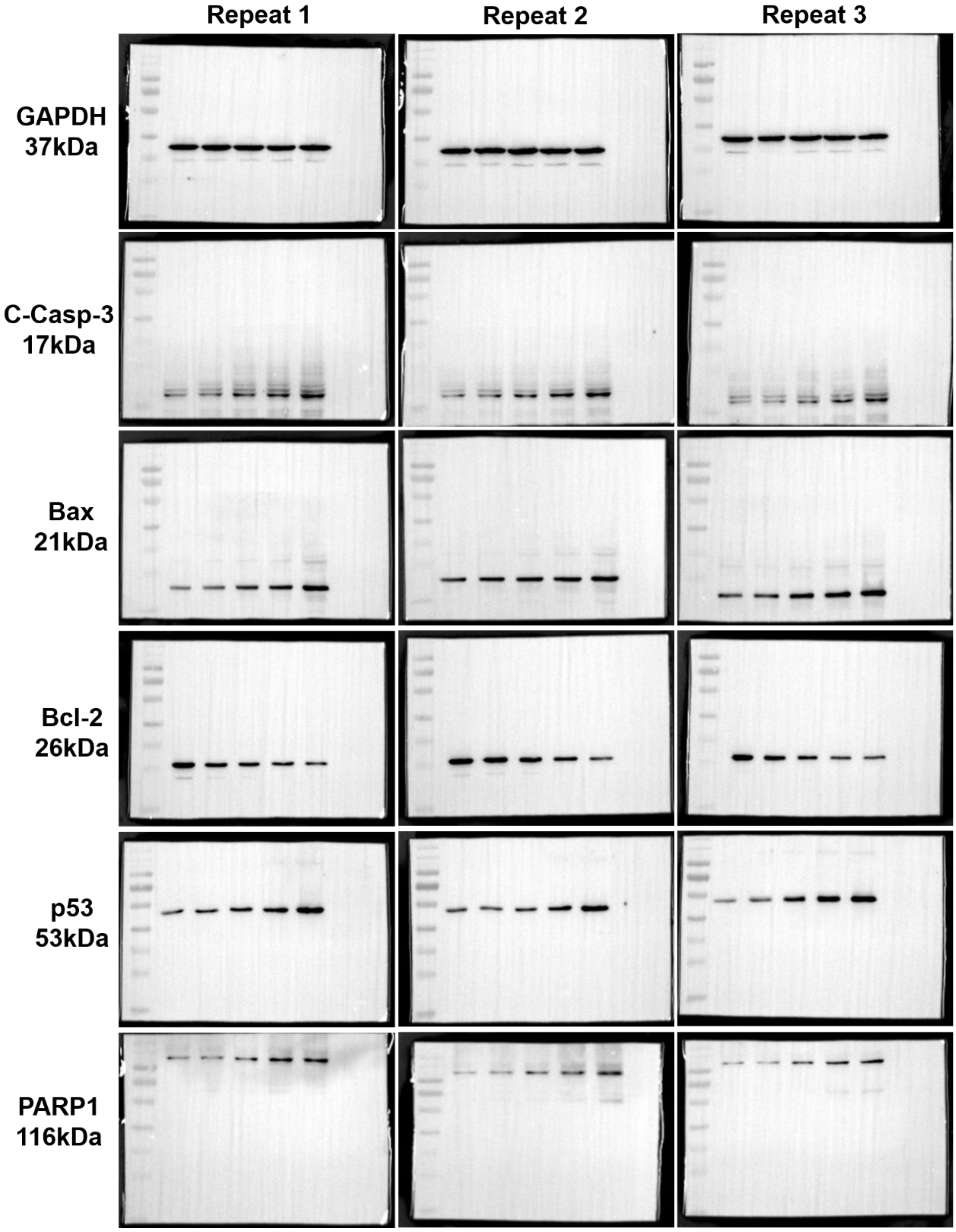

Figure S5 Original western blot for three repeats.

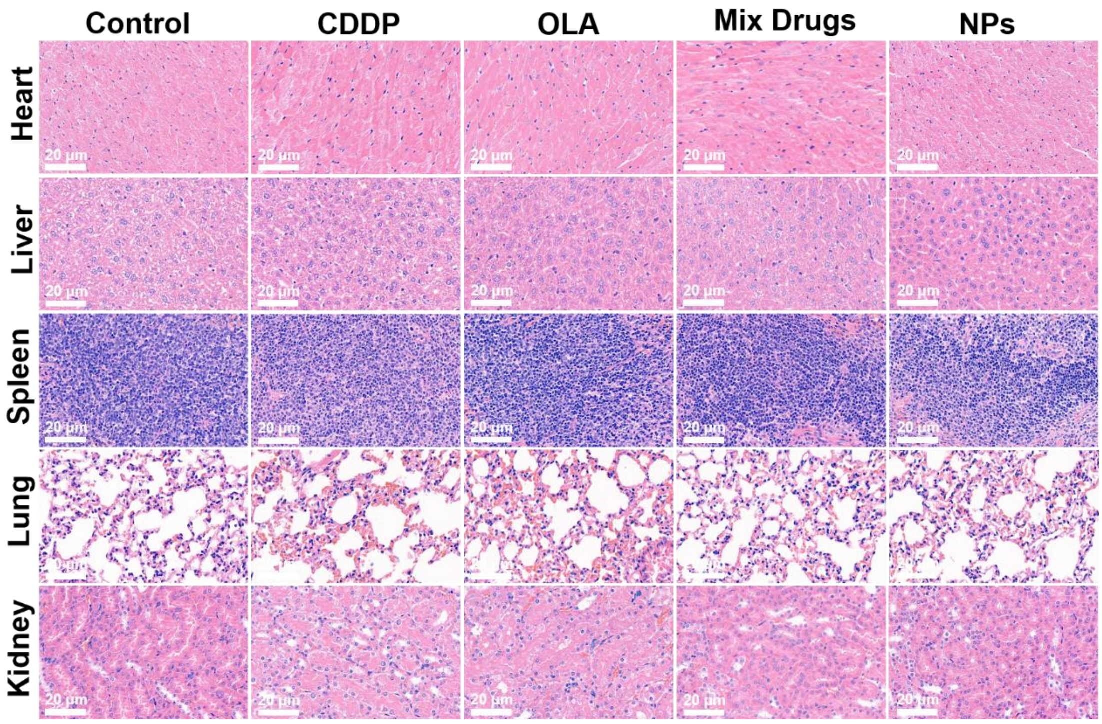

Figure S6 Histological analysis of major organs (heart, liver, spleen, lung, kidneys) from the tumor-bearing mice treated with PBS, CDDP, OLA, CDDP/OLA mixture, and CDDP-OLA NPs. All scale bars: 20 μm.
